# Ketamine versus propofol for rapid sequence induction in trauma patients: a retrospective study

**DOI:** 10.1186/s13049-021-00948-5

**Published:** 2021-09-15

**Authors:** Niklas Breindahl, Josefine Baekgaard, Rasmus Ejlersgaard Christensen, Alice Herrlin Jensen, Andreas Creutzburg, Jacob Steinmetz, Lars S. Rasmussen

**Affiliations:** 1 Department of Anaesthesia, Section 6011, Centre of Head and Orthopaedics, Rigshospitalet, University of Copenhagen, Inge Lehmanns Vej 6, Section 6011, 2100 Copenhagen, Denmark; 2 Danish Air Ambulance, Aarhus, Denmark; 3 Department of Clinical Medicine, University of Copenhagen, Copenhagen, Denmark

**Keywords:** Rapid sequence induction, Ketamine, Propofol, Intubation, Trauma, Pre-hospital

## Abstract

**Background:**

Rapid Sequence Induction (RSI) is used for emergency tracheal intubation to minimise the risk of pulmonary aspiration of stomach contents. Ketamine and propofol are two commonly used induction agents for RSI in trauma patients. Yet, no consensus exists on the optimal induction agent for RSI in the trauma population. The aim of this study was to compare 30-day mortality in trauma patients after emergency intubation prehospitally or within 30 min after arrival in the trauma centre using either ketamine or propofol for RSI.

**Methods:**

In this investigator-initiated, retrospective study we included adult trauma patients emergently intubated with ketamine or propofol registered in the local trauma registry at Rigshospitalet, a tertiary university hospital that hosts a level-1 trauma centre. The primary outcome was 30-day mortality. Secondary outcomes included hospital and Intensive Care Unit length of stay as well as duration of mechanical ventilation. We analysed outcomes using multivariable logistic regression models adjusting for age, sex, injury severity score, shock (systolic blood pressure < 90 mmHg) and Glasgow Coma Scale score before intubation and present results as odds ratios (ORs) with 95% confidence intervals.

**Results:**

From January 1st, 2015 through December 31st, 2019 we identified a total of 548 eligible patients. A total of 228 and 320 patients received ketamine and propofol, respectively. The 30-day mortality for patients receiving ketamine and propofol was 20.2% and 22.8% (*P* = 0.46), respectively. Adjusted OR for 30-day mortality was 0.98 [0.58–1.66], *P* = 0.93. We found no significant association between type of induction agent and hospital length of stay, Intensive Care Unit length of stay or duration of mechanical ventilation.

**Conclusions:**

In this study, trauma patients intubated with ketamine did not have a lower 30-day mortality as compared with propofol.

## Background

Rapid Sequence Induction (RSI) is used to facilitate emergency tracheal intubation to minimise the risk of pulmonary aspiration of stomach contents [1]. This is highly relevant in the trauma population, as these patients often are critically ill, unstable and non-fasting, and choosing the appropriate premedication will maximise the success and minimise complications [2].

Ketamine and propofol are two commonly used induction agents for RSI, yet they have very different pharmacodynamic profiles. Ketamine acts primarily through non-competitive blockade of N-Methyl-D-Aspartate (NMDA)-receptors [3] and is associated with adverse effects such as hallucinations and nightmares [4, 5]. Previous concerns related to increased myocardial ischemia or increased intracranial pressure [6–8] have recently been rejected [9–12]. Ketamine is known to increase heart rate, and blood pressure through a stimulatory effect on the sympathetic nervous system making it potentially suitable for haemodynamically unstable trauma patients [13–17]. However, similar to propofol it may cause cardiovascular collapse in haemodynamically unstable patients [14]. Propofol acts through a Gamma-AminoButyric Acid (GABA)-mediated depression of the central nervous system but is also known to have negative inotropic effects as well as vasodilation. From this perspective, propofol may not be ideal for haemodynamically unstable trauma patients [5].

As such, it seems that no ideal induction agent exists, and at present there is no consensus on which induction agent should be preferred for RSI in the trauma population [18]. Furthermore, very few studies have compared the available drugs such as ketamine and propofol [19]. Therefore, the objective of this retrospective study was to compare 30-day mortality in trauma patients after RSI facilitated emergency intubation prehospitally or within 30 min after arrival in the trauma centre using either ketamine or propofol. We hypothesised that the use of ketamine would result in a lower 30-day mortality.

## Methods

### Study design

This was a Danish, investigator-initiated, retrospective observational cohort study comparing 30-day mortality in adult trauma patients receiving an RSI facilitated intubation with ketamine or propofol. The Danish Patient Safety Authority approved the study (ID-number: 31-1522-71). According to Danish law, retrospective studies do not need approval by a Research Ethics Committee. Data management and processing was approved (Pactius ID-number: P-2020-267). Informed consent was waived given the retrospective nature of the study. The study was conducted in accordance with a pre-specified protocol and statistical analysis plan (available in Danish from the corresponding author upon request). There were no changes to study design after the study commenced. The study is reported in accordance with The Strengthening the Reporting of Observational Studies in Epidemiology (STROBE) guidelines for observational studies [20].

### Setting

We included data on adult trauma patients (≥ 18 years) admitted to Rigshospitalet in Copenhagen, Denmark, from January 1st, 2015 through December 31st, 2019. Patients from January 1st, 2015 through December 31st, 2016 were also included in a study from 2020 by Baekgaard et al. comparing in-hospital mortality in trauma patients after induction with ketamine versus other induction agents [21]. Patients were identified retrospectively through our local trauma registry. Rigshospitalet is a tertiary university hospital that hosts a level-1 equivalent trauma centre. In the Capital Region of Denmark, pre-hospital trauma care is performed by physician-staffed Mobile Emergency Care Units or physician-staffed Helicopter Emergency Medical Services and paramedic-staffed ambulances [22]. As such all intubations were performed or supervised by an anaesthesiologist.

### Inclusion and exclusion criteria

Patients were eligible for inclusion if they had been emergently intubated using either ketamine or propofol prehospitally or within 30 min after arrival to the trauma centre. Patients were excluded if no induction agent had been used, if other inductions agents than ketamine or propofol had been used, if it was unclear if ketamine or propofol had been used as induction agent, if the patients had a “no code”/Do-Not-Resuscitate (DNR) order, or they were secondary transfers from other hospitals. In cases where both ketamine and propofol had been used within approximately 15 min prior to emergency intubation and it was unclear which had been used as an induction agent, patients were categorised as receiving ketamine.

### Data collection

Data were extracted from patients’ medical records. This included sex, age, Body Mass Index, trauma mechanism (penetrating or blunt), Probability of Survival Score, Injury Severity Score (ISS), intubation location, type and dose of induction agent (ketamine or propofol), systolic blood pressure (SBP) and heart rate before and after intubation, shock prior to intubation (SBP below 90 mmHg), the presence of traumatic brain injury, injuries to the thorax, abdomen/pelvis, extremities or spine, arterial oxygen saturation and Glasgow Coma Scale (GCS) score before intubation, and the use of vasopressors and neuromuscular blockers. All vital signs before intubation were collected before administration of the induction agent.

### Sample size

We anticipated a 30-day mortality in the ketamine group of 20% versus 30% in the propofol group, equivalent to the findings by Baekgaard et al. from 2020 [21]. A sample size of 586 patients (293 in each group) would provide a power of 80% at a two-sided alpha level of 0.05 in detecting this difference. We anticipated around 150 adult trauma patients per year to be eligible for this study. We did, however, also anticipate the retrospective data to be incomplete, which is why we decided to include data over a 5-year period.

### Outcomes

The aim of this study was to compare trauma patients after emergency intubation prehospitally or within 30 min after arrival in the trauma centre using either ketamine or propofol for RSI. The primary outcome was 30-day mortality. Secondary outcomes included hospital and Intensive Care Unit (ICU) length of stay as well as duration of mechanical ventilation.

### Statistical analysis

Baseline characteristics are presented separately for each group and for the total trial population. Categorical variables are presented as frequencies (counts and percentages) and compared using chi-squared test or Fisher’s exact test as appropriate. Numerical variables are presented as medians with interquartile ranges (IQR) and compared using Student’s T-test or Wilcoxon–Mann–Whitney test as appropriate. There was no imputation of missing data.

The primary outcome was analysed using multivariable logistic regression models and the secondary outcomes were analysed using multivariable linear regression models both adjusted for age, sex, ISS, shock, and GCS score before intubation. Results are presented as odds ratios (ORs) with 95% confidence intervals for the logistic regression analyses and P-values for the linear regression analyses.

In a secondary analysis we excluded patients if both ketamine and propofol had been used within 15 min in relation to emergency intubation. We considered *P*-values < 0.05 as statistically significant.

All analyses were performed using R Studio version 3.5.3 [23].

## Results

### Study population

**Fig. 1:**
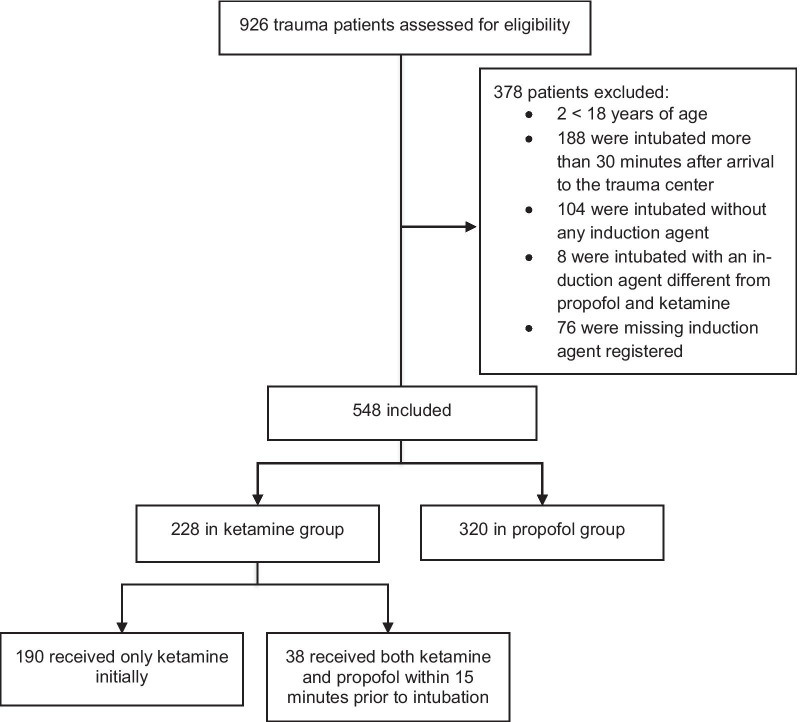
Flowchart of screening, exclusion and inclusion

In the five-year study period, a total of 926 intubated trauma patients were admitted and assessed for eligibility. We excluded 378 patients (Fig. 1). As a result, a total of 548 patients were included in the analyses: 190 (34.7%) and 320 (58.4%) patients received ketamine or propofol, respectively. A total of 38 (6.9%) patients received both ketamine and propofol within 15 min prior to intubation.

### Baseline characteristics of the study population

Most patients were male (75%), median [IQR] age was 50 years [33.0–65.1], ISS and probability of survival score were 25.0 [17.0–34.0] and 32.6% [5.7–79.7%], respectively. Most patients were intubated prehospitally (79.9%). The median [IQR] GCS score before intubation was 6.0 [3.0–10.0] and 33.8% of patients had SBP less than 90 mmHg at time of intubation, and this was significantly more common in the ketamine group (*P* = 0.003) (Table 1).

**Table 1:** Comparison of ketamine and propofol as induction agents for RSI in adult trauma patients (N = 548)

	Ketamine group (N = 228)	Propofol group (N = 320)	*P*-value
Male, N (%)	162 (71.1)	249 (77.8)	0.07^†^
Age (years), median [IQR]	48.5 [33.3, 61.5]	51.2 [32.8, 68.2]	0.08^‡^
Body Mass Index (kg per m^2^), mean (SD)	24.2 [21.4, 27.4]	24.2 [22.0, 26.5]	0.62^‡^
Missing	113	171	
ISS, median [IQR]	26.5 [19.8, 36.0]	25.0 [17.0, 29.0]	0.001*^‡^
Missing	24	75	
PSS, median [IQR]	0.4 [0.2, 0.9]	0.3 [0.0, 0.7]	< 0.001*^‡^
Missing	2	2	
Trauma mechanism, N (%)			0.002*^†^
Blunt	195 (85.5)	284 (88.8)	
Penetrating	23 (10.1)	10 (3.1)	
Burn	3 (1.3)	3 (0.9)	
Other	7 (3.1)	23 (7.2)	
Arrival mode, N (%)			0.047*^†^
Physician staffed ambulance	159 (69.7)	255 (79.7)	
Physician staffed helicopter	68 (29.8)	64 (20.0)	
Missing	1	1 (0.3)	
Known associated injuries, N (%)			
TBI	112 (49.1)	184 (57.5)	0.14^†^
Thorax	129 (56.6)	87 (27.2)	< 0.001*^†^
Abdomen/Pelvis	85 (37.3)	50 (15.6)	< 0.001*^†^
Extremities	110 (48.2)	74 (23.1)	< 0.001*^†^
Spine	82 (36.0)	68 (21.2)	< 0.001*^†^
Intubation location, N (%)			0.26^†^
Pre-hospital	177 (77.6)	261 (81.6)	
Trauma centre	51 (22.4)	59 (18.4)	
Drug dose (mg), median [IQR]	75 [50, 100]	100 [80, 150]	NA
Missing	110	105	
Vital signs before intubation, median [IQR]			
SpO_2_ (%)	96 [88, 100]	98 [90, 100]	0.06^‡^
Missing	119	138	
HR (BPM)	102 [90, 122]	95 [75, 105]	< 0.001*^‡^
Missing	117	135	
SBP (mmHg)	121 [100, 150]	130 [110, 160]	0.02*^‡^
Missing	122	143	
GCS	7 [3, 12]﻿	5 [3, 9]	0.001*^‡^
Missing	6	8	
Shock before intubation^§^, N (%)	93 (40.8)	92 (28.8)	0.003*^†^
Vital signs after intubation, median [IQR]			
HR (BPM)	104 [87, 125]	94 [75, 105]	< 0.001*^‡^
Missing	112	143	
SBP (mmHg)	115 [90, 140]	120 [100, 140]	0.22^‡^
Missing	113	136	
Change in SBP (mmHg), median [IQR]	−10 [−25, 10]	−15 [−31.5, 0]	0.06^‡^
Missing	135	164	
Neuromuscular blocking agent, N (%)			0.09^†^
* Suxamethonium*	161 (70.6)	198 (61.9)	
* Rocuronium*	33 (14.5)	67 (20.9)	
* SuxamethoniumandRocuronium*	20 (8.8)	33 (10.3)	
* Noneuromuscularblockingagent*	14 (6.1)	22 (6.8)	
Use of vasopressor, N (%)	29 (12.7)	65 (20.3)	0.04*^†^
Missing	63 (27.6)	70 (21.9)	
Outcomes			
30-day mortality, N (%)	46 (20.2)	73 (22.8)	0.46^†^
Hospital length of stay (days), median [IQR]	12 (3, 23)	6 (1, 15.2)	0.01*^‡^
ICU stay (days), median [IQR]	3 (1, 13)	1 (1, 8)	0.09^‡^
Mechanical ventilation (hours), median [IQR]	29.9 (9.6, 167.2)	20.4 (4.9, 103)	0.68^‡^

Comparison of patients in ketamine group (N = 228) and propofol group (N = 320)

*BPM* beats per minute, *GCS* Glasgow Coma Scale, *HR* heart rate, *ICU* intensive care unit, *IQR* interquartile range, *ISS* Injury Severity Score, *PSS* probability of survival score, *RSI* Rapid Sequence Induction, SpO_2_ arterial oxygen saturation, *SBP* Systolic Blood Pressure; *SD* Standard Deviation, *TBI* Traumatic Brain Injury

“Missing” denotes the number of patients with missing information in the field in question

^§^Shock: SBP before intubation < 90 mmHg

*Statistically significant: P < 0.05

^†^Pearson’s Chi-squared test

^‡^Linear Model ANOVA

### Outcomes

The 30-day mortality for patients intubated with ketamine (N = 228) compared to patients intubated with propofol (N = 320) was 20.2% and 22.8%, respectively (unadjusted *P* = 0.46). OR for 30-day mortality when intubated with propofol in the logistic regression analysis adjusted for age, sex, ISS, shock, and GCS score before intubation was 0.98 [0.58–1.66], *P* = 0.93 (Table 2).

**Table 2:** Logistic regression analysis of 30-day mortality for intubated trauma patients

	Adjusted odds ratio (95% Confidence Interval)	*P*-value
Ketamine versus propofol *(reference) primary analyses*	0.98 [0.58, 1.66]	0.93
Sex, male	1.08 [0.61, 1.95]	0.79
Age	1.05 [1.04, 1.07]	< 0.001*
ISS	1.02 [0.99, 1.04]	0.12
SBP before intubation < 90 mmHg	1.46 [0.43, 4.54]	0.53
GCS before intubation	0.84 [0.78, 0.91]	< 0.001*
Ketamine versus propofol *(reference) secondary analyses*	1.00 [0.58, 1.74]	0.99
Sex, male	1.19 [0.66, 2.20]	0.56
Age	1.05 [1.03, 1.07]	< 0.001*
ISS	1.02 [0.99, 1.04]	0.058
SBP before intubation < 90 mmHg	1.63 [0.47, 5.24]	0.42
GCS before intubation	0.85 [0.78, 0.92]	0.0002*

30-day mortality (primary outcome) for the primary and secondary analyses presented as odds ratios and 95% confidence intervals adjusted for sex, age, ISS, SBP and GCS before intubation. In the secondary analysis we excluded patients if both ketamine and propofol had been used within approximately 15 min prior to emergency intubation, and it was unclear which one had been used as induction agent

*GCS* Glasgow Coma Scale, *ISS* Injury Severity Score, *SBP* Systolic Blood Pressure

*Statistically significant: P < 0.05

We found no significant association between type of induction agent and hospital length of stay, ICU length of stay or duration of mechanical ventilation (*P* = 0.43, *P* = 0.68, *P* = 1.00, respectively) (Table 3).

**Table 3:** Multivariable linear regression analyses of secondary outcomes for intubated trauma patients

	*P*-value for hospital LOS	*P*-value for ICU LOS	P-value for duration of mechanical ventilation
Ketamine versus propofol (reference) primary analyses (N = 548)	0.43	0.68	1.00
Sex, male	0.54	0.85	0.99
Age	0.007*	0.27	0.99
ISS	0.85	0.0003*	1.00
SBP before intubation < 90 mmHg	0.88	0.97	1.00
GCS before intubation	< 0.001*	0.45	0.99
Ketamine versus propofol (reference) secondary analyses (N = 510)	0.67	0.89	0.79
Sex, male	0.34	0.29	0.58
Age	0.005*	0.003*	0.04*
ISS	0.039*	0.002*	0.2
SBP before intubation < 90 mmHg	0.59	0.22	0.94
GCS before intubation	0.24	0.1	0.07

Multivariable linear regression analyses of secondary outcomes (hospital length of stay, ICU length of stay and duration of mechanical ventilation) for the primary (N = 548) and secondary analyses (N = 510) presented as *P*-values adjusted for sex, age, ISS, SBP and GCS before intubation. In the secondary analysis we excluded patients if both ketamine and propofol had been used within approximately 15 min prior to emergency intubation, and it was unclear which one had been used as induction agent

*GCS* Glasgow Coma Scale, *ICU* Intensive Care Unit, *ISS* Injury Severity Score, *LOS* Length of Stay, *SBP* Systolic Blood Pressure

*Statistically significant: P < 0.05

Excluding patients receiving both ketamine and propofol in the secondary analyses did not alter the result (Tables 2 and 3).

## Discussion

In this retrospective cohort study, we found no difference between 30-day mortality for trauma patients intubated with RSI using ketamine compared to propofol. There were statistically significant differences between the ketamine and propofol group regarding systolic blood pressure and GCS score before intubation. However, we did not observe any significant haemodynamic difference between the two agents in terms of SBP after intubation or change in SBP after intubation. No significant difference regarding hospital and ICU length of stay or duration of mechanical ventilation was identified either.

This study has several strengths. First, it is one of the largest studies comparing ketamine to propofol for RSI in the trauma population. Second, in the Capital Region of Denmark other aspects of trauma care such as the use of tranexamic acid, prehospital plasma, and spinal immobilisation are standardised. Both propofol or ketamine are recommended single induction agents in our trauma system, in combination with either suxamethonium, or rocuronium. In addition, an opioid, being either fentanyl or alfentanil, is usually given. However, the choice of anaesthetic technique, dosing and induction agents are based on an individual assessment made by the attending physician, and the retrospective study design does not allow for an analysis of why the particular induction agent was chosen in the specific situation. Third, the follow-up completeness regarding the primary and secondary outcomes was 100% adding reliability to our outcome assessment. However, the retrospective design of this study has several limitations. First, we did not use a strictly standardised drug protocol specifying drug dosage or indications for drug choice, and due to the retrospective design of the study we cannot comment on why the physicians chose one induction agent above the other. These aspects of trauma care did not change significantly in our pre- or in-hospital standard operating procedures for trauma care during the data collection period. Second, the timing of data collection may be inconsistent which made it difficult to describe and analyse all details. Third, in 76 (13.9% of) cases information about the type of induction agent was missing in the medical records. This introduces a bias through the﻿ possibility of missing not at random, possibly occurring when treating a critically ill patient without enough time to routinely collect information. Additionally, this study only includes patients who were admitted alive/under resuscitation to the hospital and does not consider the severely traumatised patients who were declared dead in the pre-hospital setting, as data on these patients are not recorded in the local trauma registry at Rigshospitalet which we used for data collection. Also, this study does not provide any data on the long-term outcome of trauma patients making it impossible to evaluate any significant outcomes other than in-hospital outcomes.

Even though the choice of induction agent is truly a small alteration in the trauma care pathway, Baekgaard et al. from 2020 [21] found 30-day mortality rate to be 20% and 30% for ketamine and propofol, respectively. We anticipated the same mortality rates and calculated a total sample size of 586 patients. Yet, our study showed mortality rates of 20.2% and 22.8% for ketamine and propofol, respectively, and we only included 548 patients in the designated period, 228 and 320 for ketamine and propofol, respectively. This introduces risk of type 2 error as illustrated by the wide confidence interval regarding 30-day mortality [0.58–1.66] and we cannot exclude a clinically meaningful difference.

We adjusted our analyses for sex, age, ISS, SBP and GCS before intubation. Yet, several pre-hospital factors with a significant role in 30-day mortality were not registered, e.g., ambulance response time and hospital arrival time, difficult airway predictors etc.

A recent systematic review compared studies where trauma patients were intubated in the prehospital setting or within 1 h of emergency department arrival using RSI with ketamine compared to other agents [19]. Four studies were eligible: one compared ketamine to thiopental and showed no difference in number of blood transfusions [24], and three compared ketamine to etomidate: A randomised controlled trial from 2009 showing no difference in 28-day mortality [25], a cohort study from 2015 showing no difference in hospital mortality [26], and finally an observational study from 2017 showing no difference in number of blood transfusions, hospital length of stay or in-hospital mortality [27]. Yet, these studies have several limitations, as they were rather small and therefore inconclusive. A study from 2020 by Baekgaard et al. [21] made similar findings with no difference in mortality comparing ketamine with other induction agents (propofol, etomidate, or midazolam). Finally, one recent multicentre study from 2021 reported no change in average SBP during RSI with ketamine compared to other agents in the adult trauma population [28].

Today, ketamine is considered safe and effective in the emergency department and the prehospital setting [4, 26, 29], and its use is increasing. Concerns regarding increased myocardial ischemia or increased intracranial pressure from intubation with ketamine have been rejected [9, 10], and it may actually be considered neuroprotective [30]. Yet, a randomised trial of induction agents or a prospective study with clearly documented standardised anaesthetic technique is needed, as no currently available evidence supports a specific agent used in RSI [10, 19]. This warranted trial should standardise the administration and dosage of opioids, choice of neuromuscular blocking agent and the maintenance sedation as potentially confounding factors.

## Conclusion

Trauma patients intubated with ketamine did not have a lower 30-day mortality as compared with propofol. Moreover, we did not find a difference in hospital or ICU length of stay or duration of mechanical ventilation between the two induction agents. A randomised set-up is warranted to confirm our findings.

## Data Availability

The datasets used and/or analysed during the current study are available from the corresponding author on reasonable request.

## Abbreviations

BPM: Beats Per Minute
DNR: Do-Not-Resuscitate
GCS: Glasgow Coma Scale
HR: Heart Rate
ICU: Intensive Care Unit
IQR: Interquartile Range
ISS: Injury Severity Score
LOS: Length of Stay
OR: Odds Ratio
PSS: Probability of Survival Score
RSI: Rapid Sequence Induction
SpO_2_: Arterial oxygen saturation
SBP: Systolic Blood Pressure
SD: Standard Deviation
STROBE: The Strengthening the Reporting of Observational Studies in Epidemiology
TBI: Traumatic Brain Injury
